# Seeking brightness from nature: controlled synthesis of multicolor fluorescent carbon dots from biomass

**DOI:** 10.1039/d6na00062b

**Published:** 2026-03-24

**Authors:** Liu Ding, Chihao Wang, Shouwang Kang, Zhongguo Zhao

**Affiliations:** a School of Material Science and Engineering, Shaanxi University of Technology Hanzhong 723000 Shaanxi P. R. China dingliu@snut.edu.cn; b School of Materials and Chemical Engineering, Xi'an Technological University Xi'an 710021 China

## Abstract

Fluorescent carbon dots (CDs) exhibit broad application prospects in multiple fields, such as food testing and biomedicine, owing to their abundant raw material sources, excellent biocompatibility, and superior fluorescence properties. However, the diversity of biomass raw materials poses challenges to the controllable preparation of CDs, significantly limiting their large-scale production and practical application. In this study, bamboo leaves were employed as a single precursor, and a one-step solvothermal method was adopted. By regulating the reaction temperature, precise synthesis of multi-band fluorescent CDs was achieved, successfully yielding red-fluorescent R-CDs (emitting at 680 nm), pink dual-emission fluorescent P-CDs (emitting at 480 nm/680 nm), and blue-fluorescent B-CDs (emitting at 450 nm). All synthesized CDs possess excellent optical properties, characterized by high fluorescence intensity and narrow emission bands. Notably, B-CDs demonstrate remarkable specific recognition toward tartrazine through the synergistic effect of internal filtering and static quenching. This study provides a simple and efficient technical approach for the controlled synthesis of biomass-derived CDs, further expanding their application potential in food detection, chemical analysis, and biomedicine.

## Abstract 2

Fluorescent carbon dots (CDs) exhibit broad application prospects in multiple fields, such as food testing and biomedicine, owing to their abundant raw material sources, excellent biocompatibility, and superior fluorescence properties.

## Graphical abstract

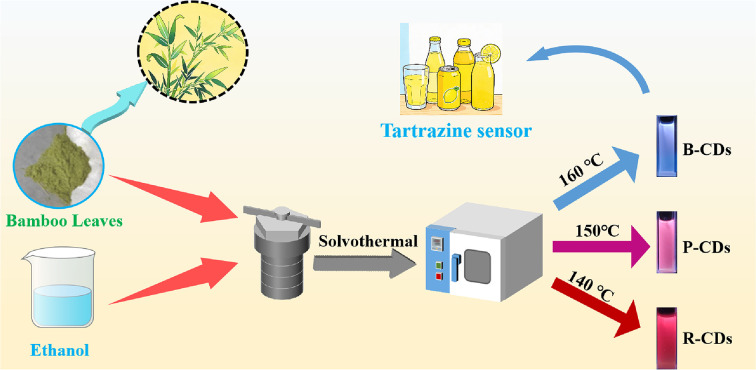

## 1 Introduction

Carbon dots (CDs), as an emerging class of carbon-based nanomaterial, demonstrate tremendous application potential across numerous fields owing to their exceptional fluorescence properties,^1–3^ low toxicity,^4,5^ excellent biocompatibility,^6^ and robust chemical stability.^7,8^ In recent years, with the progressive advancement of sustainable development concepts, green chemistry has emerged as a pivotal guiding principle in materials synthesis.

CD preparation can be broadly classified into two categories based on precursor sources: conventional chemically synthesized CDs and biomass-derived fluorescent CDs. Traditional methods typically employ organic reagents such as ethylene glycol and benzidine as precursors.^9,10^ These raw materials not only incur high costs, posing challenges to cost control, but also impose substantial environmental burden during the synthesis process. In contrast, biomass-derived fluorescent CDs utilize renewable resources including plant tissues, animal byproducts, agricultural waste, and microorganisms. These feedstocks offer distinct advantages such as abundant availability, high renewability, and excellent biocompatibility. Rich in active components like cellulose, lignin, proteins, and various trace elements, they serve as ideal carbon sources for CD synthesis.^8,11–14^

Numerous biomass-derived CDs have been reported. For instance, Chen *et al.*^8^ synthesized biomass CDs from orange peel *via* hydrothermal synthesis for modifying natural rubber. The CDs enhanced the mechanical properties and significantly improved the antioxidant characteristics of natural rubber. Ban *et al.*^11^ utilized banana as a precursor to prepare biomass CDs through hydrothermal synthesis. They combined CDs with quaternary ammonium salt chitosan and sericin to develop a composite coating for vegetable surfaces, effectively extending the shelf life of Chinese broccoli. Additionally, numerous biomass precursors like wheat straw, sugarcane, pine needles, and seed cakes have been used to synthesize CDs.^15–18^ However, despite the advantages of biomass CDs, their large-scale application faces critical technical bottlenecks: biomass feedstocks exhibit complex and highly diverse compositions, with significant variations in elemental content and functional group structures across different materials. Even within the same feedstock, factors like growth environment and harvest timing influence product properties, making precise control of key optical parameters – such as fluorescence wavelength and quantum yield – challenging and limiting controllable synthesis. The inherent uncertainty in the preparation process leads to poor reproducibility of biomass CD performance, which fails to meet the material stability requirements for practical applications. Consequently, this severely hinders the translation of biomass-derived CDs from laboratory research to industrial production and real-world utilization. Thus, developing a simple, efficient method for preparing biomass CDs with precisely tunable fluorescence properties has become an urgent and core issue in this field.

This study employs bamboo leaves as precursors to synthesize a series of biomass-derived CDs *via* a one-step solvothermal method. By varying reaction temperatures, the fluorescence wavelengths of the CDs are tuned, enabling the preparation of multicolor fluorescent CDs. Subsequently, the surface functional groups, morphology, and optical properties of the CDs were analyzed using transmission electron microscopy (TEM), X-ray photoelectron spectroscopy (XPS), Fourier transform infrared spectroscopy (FT-IR), fluorescence spectroscopy, and ultraviolet spectroscopy. Furthermore, the sensitivity and reliability of CDs for pigment detection were validated by investigating the correlation between different tartrazine concentrations in CDs and the degree of fluorescence quenching. This research not only advances the controllable preparation technology of biomass-derived CDs but also lays a crucial foundation for their large-scale practical applications.

## 2 Results and discussion

### 2.1 Optimization of reaction conditions

At the initial stage of preparation, appropriate biomass precursors must be selected for the synthesis of CDs. Using bamboo leaves, weeping willow, camellia, and peach blossoms as precursors, biomass fluorescent CDs with different fluorescent colors were obtained *via* the solution thermal method after reacting at 140 °C for 8 hours. As shown in Fig. S1a, under reaction conditions of 140 °C and 8 hours, the CD solution derived from bamboo leaves exhibited the strongest fluorescence intensity with a red emission. In contrast, the CD solutions from camellia and peach blossom precursors displayed blue fluorescence with weaker intensity. Therefore, bamboo leaves were selected as the precursor. To obtain CDs with superior performance for subsequent characterization and application, the reaction temperature and duration of CDs were controlled. The fluorescence emission spectra of CDs at different temperatures are shown in Fig. S1b. At a reaction time of 8 h and temperatures of 140 °C, 150 °C, and 160 °C, the CDs exhibited red, pink, and blue fluorescence colors, respectively. As shown in Fig. S1b, increasing the temperature from 140 °C to 160 °C progressively enhances the fluorescence intensity of the short-wavelength absorption peak while diminishing that of the long-wavelength absorption peak. The long-wavelength peak exhibits a blue shift tendency, causing the solution's fluorescence color to transition from red to blue. At a reaction temperature of 160 °C, the fluorescence color of the CD solution was observed to change over reaction times of 4 h, 6 h, 8 h, and 12 h, as shown in Fig. S1c. As the reaction time increased from 4 h to 12 h, the fluorescence color of the CD solution shifted from red to blue. As the reaction time increased from 4 h to 12 h, the fluorescence intensity of the short-wavelength absorption peak gradually increased, while that of the long-wavelength absorption peak gradually decreased.

**Scheme 1:**
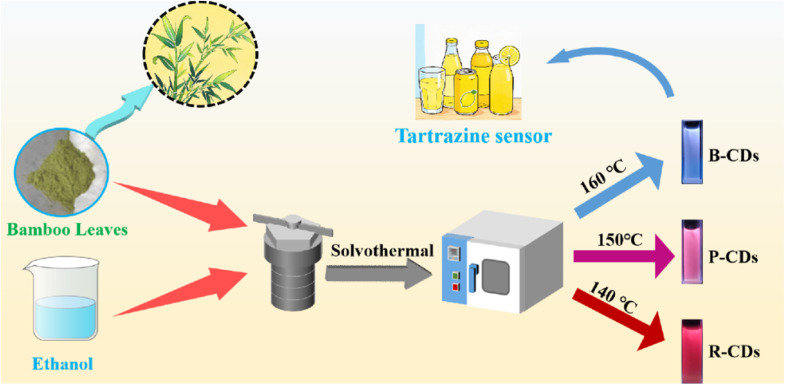
Schematic diagram of CDs prepared from bamboo leaves.

In summary, CDs were synthesized using bamboo leaves as precursors with a reaction time of 8 h and at reaction temperatures of 140 °C, 150 °C, and 160 °C. They were named R-CDs, P-CDs, and B-CDs according to their fluorescence colors (Scheme 1).

### 2.2 Structural characterization analysis of CDs

**Fig. 1:**
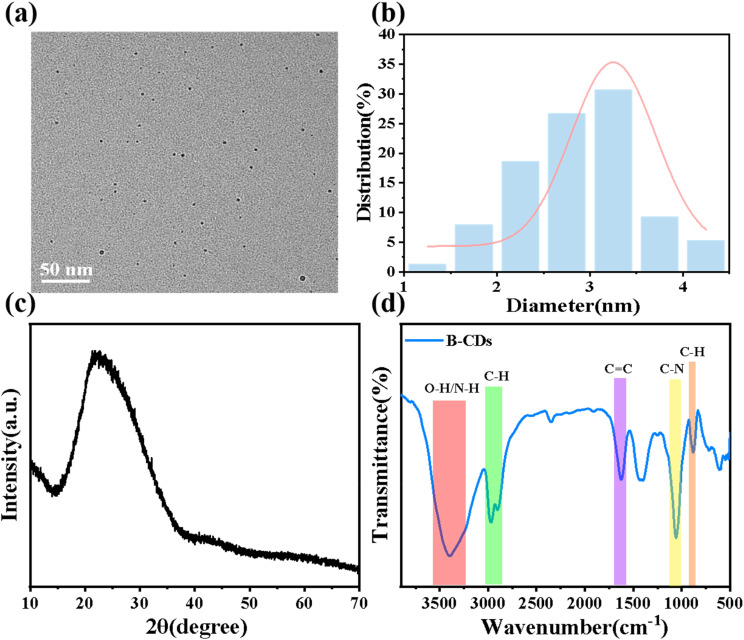
(a) TEM image of B-CDs. (b) Particle size distribution of B-CDs. (c) XRD pattern and (d) FT-IR spectrum of B-CDs.

A comprehensive analysis of the CDs regarding their structure and surface morphology was conducted *via* high-resolution transmission electron microscopy (HR-TEM), X-ray photoelectron spectroscopy (XPS), X-ray diffraction (XRD), and Fourier transform infrared (FT-IR) spectroscopy. The morphology of CDs was analyzed *via* HR-TEM. As shown in Fig. 1b, the particle size distribution indicates that B-CDs range from 1.4 to 4.6 nm in diameter, with an average size of 2.85 ± 0.06 nm. TEM images reveal that B-CDs exhibit spherical, monodisperse nanoparticles with negligible agglomeration. Fig. 1c displays the XRD pattern of B-CDs, with a diffraction peak at 22.3° corresponding to the (002) plane. These data indicate that B-CDs possess an amorphous carbon structure similar to graphite carbon.^19^ FT-IR analysis investigated the structures and functional groups of B-CDs. As shown in Fig. 1d, the FT-IR spectra of B-CDs reveal absorption peaks at 3405 cm^−1^ corresponding to O–H/N–H bond stretching vibrations.^20^ Absorption peaks in the 2859–3027 cm^−1^ region indicate C–H stretching vibrations,^21^ while the absorption peak near 1635 cm^−1^ is attributed to C=C bond stretching.^22^ The characteristic peak at 1054 cm^−1^ corresponds to C–N stretching, and the peak at 892 cm^−1^ represents the out-of-plane bending vibration of C–H bonds in alkenes or aromatics.^23^ B-CDs exhibit smooth peak shapes, likely due to the solution environment promoting intermolecular interactions such as hydrogen bonding, which may influence peak intensity and shape.

**Fig. 2:**
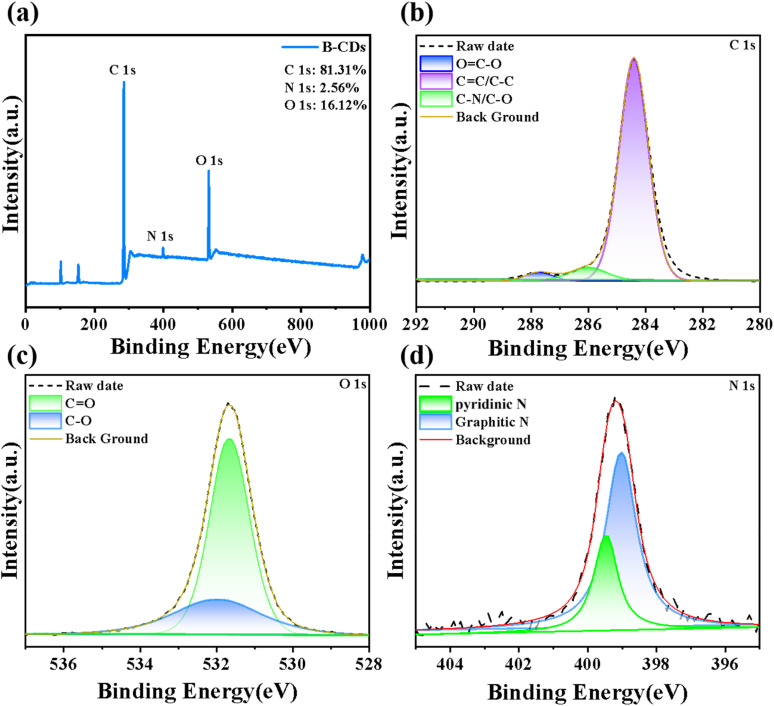
(a) XPS spectrum of B-CDs; XPS patterns of (b) O 1s, (c) N 1s and (d) C 1s.

X-ray photoelectron spectroscopy (XPS) was utilized to examine the accurate elemental composition of B-CDs. As illustrated in Fig. 2a, the full XPS spectrum presented three characteristic peaks at 284.41 eV, 399.18 eV and 531.67 eV, revealing that B-CDs consist of C, N, and O elements with atomic percentages of 81.31%, 2.56% and 16.12%, respectively. The high-resolution C 1s spectrum (Fig. 2b) showed three distinct peaks, which were confirmed to be associated with C–C/C=C, C–O, and C=O functional groups.^24^ In addition, the high-resolution O 1s XPS spectrum (Fig. 2c) demonstrated two different chemical environments of oxygen: a peak corresponding to C=O was detected at 531.7 eV, and another peak assigned to C–O appeared at 532.1 eV.^25^ The N 1s spectra for B-CDs display two component peaks at 399.1 and 399.5 eV, which correspond to pyridinic N and graphitic N, respectively.^26^ XPS spectra confirm that the surface of B-CDs is covered with a series of functional groups, including nitrogen-containing groups.

### 2.3 Optical properties of CDs

**Fig. 3:**
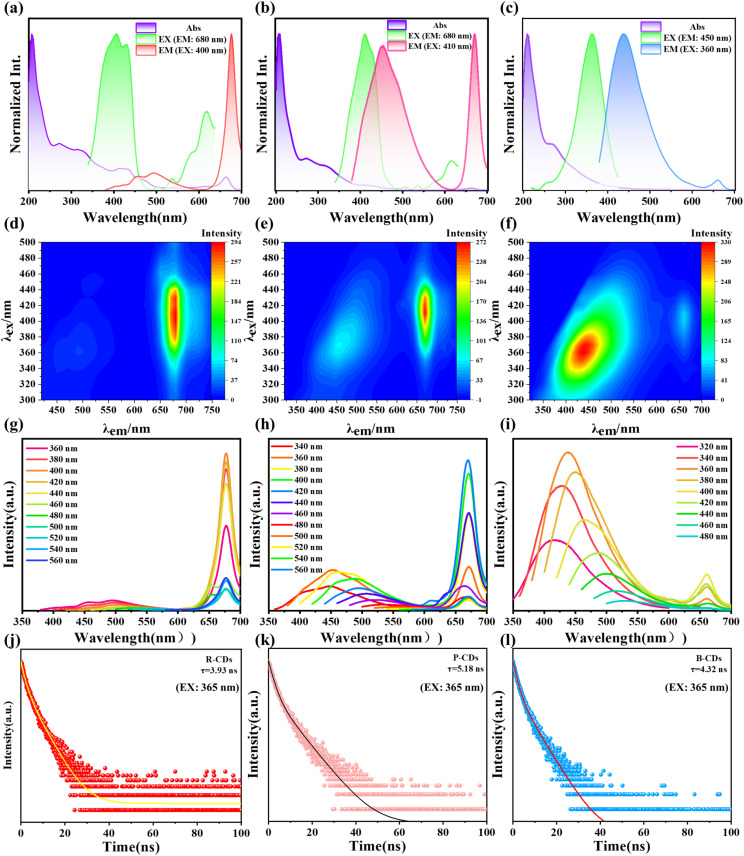
UV-Vis absorption, FL excitation (Ex) and emission (Em) spectra of T-RCD powder (a), T-PCD powder (b) and T-BCD powder (c). Excitation–emission matrix spectra of T-RCD powder (d), T-PCD powder (e) and T-BCD powder (f). Fluorescence excitation spectra of T-RCDs (g), T-PCDs (h) and T-BCDs (i) under different excitation conditions. Fluorescence decay curves of as-prepared T-RCD (j), T-PCD (k) and T-BCD (l) solutions and powders.

To investigate the optical properties of biomass-derived fluorescent R-CDs, P-CDs and B-CDs, their optical characteristics were investigated using ultraviolet-visible (UV-Vis) absorption spectroscopy, fluorescence spectroscopy, and fluorescence decay curves. As shown in Fig. 3a–c, the strong absorption peak near 230 nm originates from π–π* transitions in C=C bonds.^27^ A minor absorption peak near 670 nm likely stems from chlorophyll and its degradation products. The optimal excitation peak is located at 400 nm, with a corresponding emission peak near 680 nm. Fig. 3b shows that P-CDs exhibit two emission peaks at 450 nm and 680 nm, with both peaks having nearly identical intensities, resulting in a pink color for the CD solution. Fig. 3c reveals a prominent absorption peak near 230 nm for B-CDs, arising from π–π* transitions in C=C bonds.^28,29^ Its main emission peak is at 450 nm, with optimal excitation near 360 nm. To further investigate the optimal excitation and emission wavelengths for R-CDs, P-CDs and B-CDs, excitation–emission matrix diagrams were obtained as shown in Fig. 3d–f. The optimal PL emission peaks for R-CDs, P-CDs and B-CDs under optimal excitation at 400 nm, 410 nm, and 360 nm, respectively, were observed at 680 nm, 480 nm/680 nm, and 450 nm. The fluorescence emission curves of R-CDs, P-CDs and B-CDs under different excitations were further investigated. As shown in Fig. 3g, R-CDs exhibit single-peak emission at 680 nm, demonstrating excitation-independent behavior as the excitation wavelength increases. In contrast, P-CDs display dual-peak emission, with fluorescence intensity gradually decreasing and exhibiting a red-shift trend as the excitation wavelength increases from 360 nm to 560 nm, indicating excitation-dependent characteristics. B-CDs exhibit bimodal emission dominated by short-wavelength absorption peaks, rendering the solution blue. As the excitation wavelength increases from 320 nm to 480 nm, the fluorescence intensity first increases then decreases while exhibiting a red shift trend, demonstrating excitation dependence.

Then, the time-resolved PL decay curves of these samples were measured under 365 nm excitation, and the results are shown in Fig. 3j–l. The average fluorescence lifetime of CDs was calculated using eqn (1) and (2):1
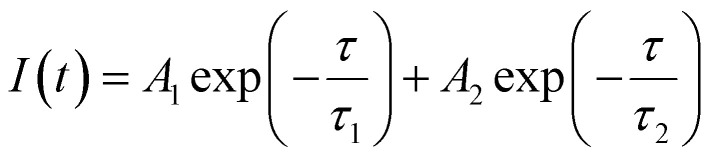
2
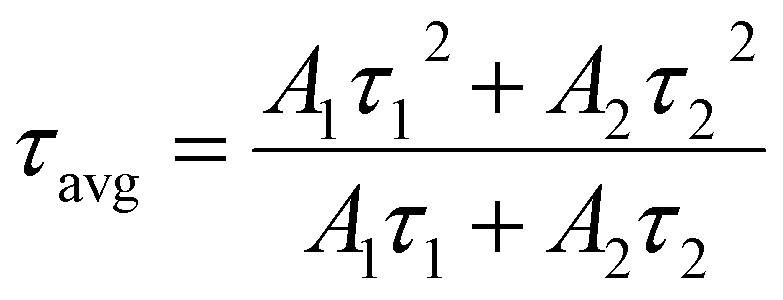


Fluorescence lifetime fitting was performed and the average fluorescence lifetimes of R-CDs, P-CDs and B-CDs were determined to be 1.08 ns, 0.88 ns and 0.29 ns, respectively. The fluorescence quantum yields (QYs) for B-CDs, P-CDs and R-CDs are 32.28%, 28.16% and 10.94%, respectively.

## 3 B-CD recognition of lemon pigments

The stability of B-CDs was investigated by measuring their fluorescence emission spectra at different pH values. As shown in Fig. S2, the emission intensity increased with rising pH when excited at 365 nm. From pH 4 to 7, the fluorescence intensity of B-CDs gradually increased but at a small rate, tending toward relative stability. At pH > 7, the fluorescence intensity rapidly decreases, reaching a maximum at pH = 7. This decline stems from potential protonation and deprotonation under alkaline conditions, where interactions between the –OH and –COOH functional groups of CDs induce aggregation, leading to fluorescence quenching.^30^

**Fig. 4:**
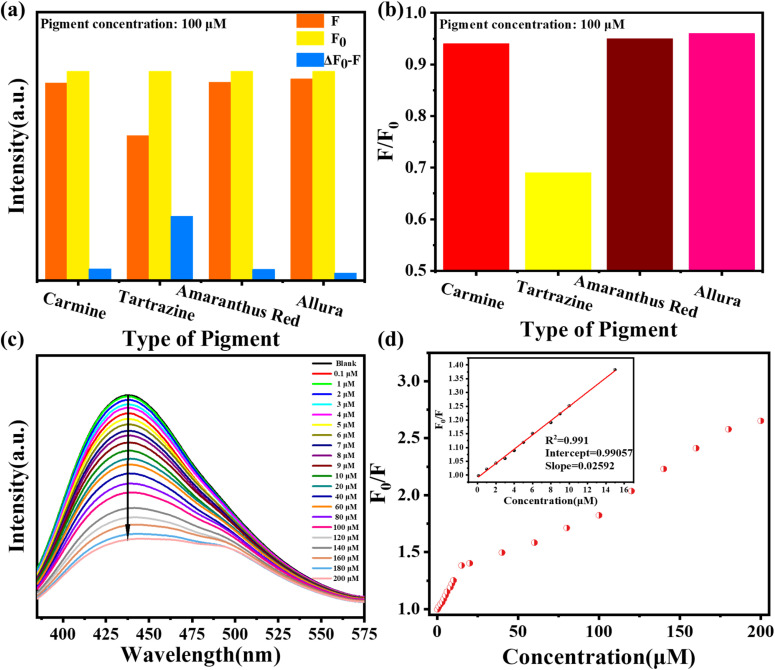
(a and b) Interference of different types of pigments in the B-CD system. (c) Fluorescence spectra of B-CDs with various concentrations (0.1–200 µM) of tartrazine. (d) Relationship of fluorescence ratio (*F*_0_/*F*) with concentration of tartrazine (the illustration is a linear diagram).

Further exploration of the potential applications of B-CDs in pigment sensing was carried out. Four pigments – carmine, tartrazine, allura and amaranthus red – were added to B-CDs at a concentration of 100 µM. As shown in Fig. 4a, among the food pigments tested, only tartrazine significantly quenched B-CDs. The presence of functional groups such as amino, carboxyl, and hydroxyl groups on the B-CD surface may form complexes with sulfonic acid or hydroxyl groups on the azo dye surface, leading to quenching. Other pigments possess fewer surface functional groups, resulting in the prepared B-CDs exhibiting optimal quenching effects for tartrazine. Tartrazine is one of the most widely used dyes in various foods, including dietary supplements, beverages, candies, jellies, sauces and baked goods, imparting a lemon-yellow color. Consequently, subsequent studies focused on tartrazine.

To determine the detection limit and quantification limit of B-CDs, a further study was conducted over a concentration range from 0 to 200 µM (Fig. 4c). A decrease in the emission intensity of B-CDs was observed. A linear relationship was observed when the concentration of tartrazine increased from 0.1 to 15 µM (Fig. 4b). The limit of detection (LOD) and limit of quantification (LOQ) calculated based on the Stern–Volmer plot (Fig. 4d) were 0.115 µM and 0.347 µM, respectively. The B-CD-based fluorescence analysis method is simple to operate, requires no organic solvents, and offers a lower detection limit. To date, several sensors for tartrazine fluorescence detection have been developed, as summarized in Table 1.

**Table 1:** Comparison of different methods for the detection of tartrazine

Detection method	Detection limit (µM)	LOD (µM)	Reference
Fluorescence ([Eu(ppda)(bpdc)_0.5_(C_2_H_5_OH)·(H_2_O)])	0.000741–0.0493	0.0987	31
Fluorescence (CS-CQDs)	0–70	0.48	32
Fluorescence (CQDs)	0–500	0.09	30
Electrochemistry (Ni_2_P@f-CNF)	0.01–1875	0.011	33
Fluorescence (K-CDs)	0.1–70	0.023	34
Fluorescence (B-CDs)	0.1–200	0.115	Our work

**Fig. 5:**
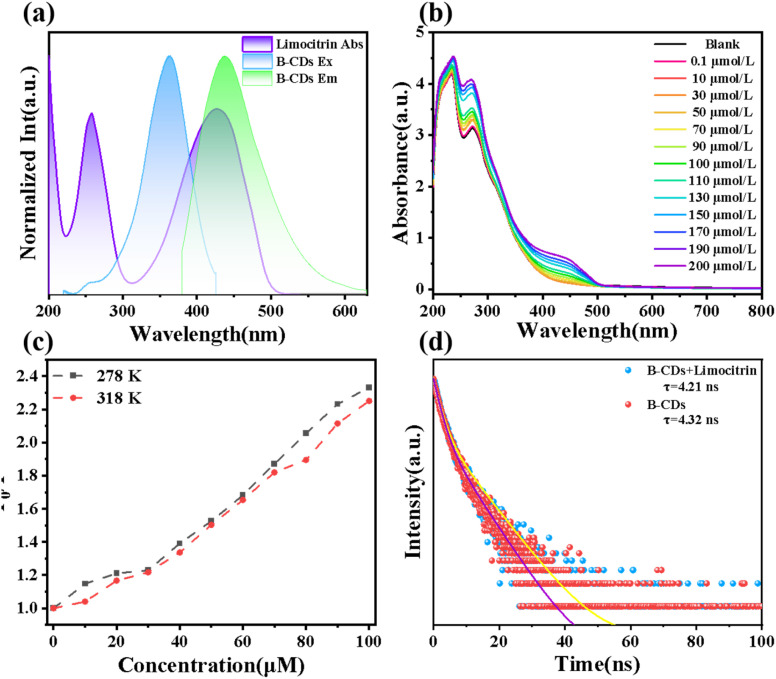
(a) Overlapping graph of the UV spectrum of tartrazine, the excitation and emission spectra of B-CDs. (b) Effects of concentrations of tartrazine on the UV spectra. (c) Stern–Volmer fitting graph of *F*_0_/*F versus* tartrazine at different temperatures. (d) Fluorescence decay curves of B-CDs with and without the addition of 20 µM tartrazine.

Further investigation into the quenching mechanism between B-CDs and tartrazine was conducted. As shown in Fig. 5, multiple spectroscopic measurements were employed to elucidate the interaction mechanism between the surface functional groups of B-CDs and tartrazine molecules. The absorption spectrum of tartrazine, spanning 300–500 nm, largely overlaps with the emission spectrum of B-CDs, indicating that fluorescence quenching is primarily due to an internal filtering effect (IFE) between the two (Fig. 5a). Tartrazine cannot effectively shield the emission radiation of B-CDs but may absorb energy at the excitation wavelength. This absorption may contribute to reduced fluorescence intensity, suggesting that IFE could account for the observed quenching. As shown in Fig. 5b, UV-Vis absorption spectra reveal a new absorption peak at 440 nm for B-CDs with increasing tartrazine content, while absorption peaks in the 300–500 nm range gradually increase with tartrazine concentration. These results further suggest that the system follows a static quenching mechanism.^35^ Additionally, Stern–Volmer plots at different temperatures (278 K and 318 K) indicate that the slope of the curve decreases with increasing temperature (Fig. 5c). This reflects that elevated temperatures reduce the stability of the complex, consistent with the static quenching mechanism. Fig. 5d displays the fluorescence lifetime of B-CDs in the absence and presence of tartrazine. The study found that the fluorescence lifetime of B-CDs was 4.32 ns, while it was 4.21 ns in the presence of tartrazine. This indicates that the lifetime of B-CDs remains virtually unchanged in the presence of tartrazine, further confirming that the quenching is caused by a static effect. In summary, it can be inferred that the fluorescence quenching of B-CDs by tartrazine results from the combined effects of internal filtering and static quenching.

## 4 Conclusions

In summary, high-performance biomass-derived fluorescent CDs were successfully synthesized from bamboo leaves *via* a one-step solvothermal reaction. By modulating the reaction temperature, the fluorescence wavelengths of the CDs were tuned, yielding three types of stable fluorescent CDs: red-fluorescent R-CDs (emitting at 680 nm), pink-fluorescent P-CDs (emitting at 480 nm/680 nm), and blue-fluorescent B-CDs (emitting at 450 nm). Structural characterization confirmed that R-CDs, P-CDs and B-CDs all possessed uniform size distribution. These CDs featured a conjugated carbon core dominated by C=C double bonds, while their surfaces were densely functionalized with abundant hydrophilic groups, including hydroxyl (–OH) and carbonyl (C=O) moieties. Moreover, the as-synthesized CDs exhibited exceptional optical properties, characterized by high fluorescence quantum yields, intense emission intensity, and narrow spectral bandwidths. Taking advantage of their unique structural features and superior optical performance, the CD aqueous solutions were successfully applied to the sensitive detection of tartrazine, a widely used synthetic pigment. Notably, for B-CDs, a strong linear correlation (*R*^2^ = 0.98432) was observed between the tartrazine concentration and the fluorescence quenching ratio (*F*/*F*_0_), demonstrating excellent sensing capabilities. This finding highlights the great potential of these biomass-derived CDs for practical applications in food safety monitoring and biomedical sensing.

## Author contributions

The manuscript was written through contributions of all authors. All authors have given approval to the final version of the manuscript.

## Conflicts of interest

The authors declare that they have no conflict of interest.

## Supplementary Material

NA-OLF-D6NA00062B-s001

## Data Availability

The data supporting this article have been included within the text. Supplementary information (SI) is available. See DOI: https://doi.org/10.1039/d6na00062b.
