# Supplementary material for: Seeking brightness from nature: controlled synthesis of multicolor fluorescent carbon dots from biomass
Source: Nanoscale Adv. 2026 Mar 24;8(9):2977–84. doi: 10.1039/d6na00062b (PMC13066824; doi:10.1039/d6na00062b)
Supplement: NA-008-D6NA00062B-s001 [file NA-008-D6NA00062B-s001.pdf]

Supplementary Information (SI) for Nanoscale Advances.
This journal is © The Royal Society of Chemistry 2026

Seeking Brightness from Nature: Controlled Synthesis of Multicolor Fluorescent Carbon Dots from Biomass

Liu Ding[a, b*], Chihao Wang[a], Shouwang Kang[a] and Zhongguo Zhao[a]

AUTHOR ADDRESS

a. School of Material Science and Engineering, Shaanxi University of Technology, Hanzhong 723000, Shaan xi, P. R. China.

b. School of Materials and Chemical Engineering, Xi`an Technological University, Xi`an 710021, China.

*Corresponding authors at: School of Material Science and Engineering, Shaanxi University of Technology, Hanzhong 723000, Shaan xi, P. R. China.

E-mail addresses: dingliu@snut.edu.cn

Supplementary Information

Materials

Bamboo leaves are sourced from Hanzhong City, Shaanxi Province, China. Carmine, tartrazine, allura, and amaranthus red were bought from Sace Chemical Technology Co., LTD (Shanghai, China). Ethanol was bought from Anhui Zesheng Senrise Technology Co., Ltd (Anhui, China). All reagents were of analytical grade and used directly without further purification. Deionized (DI) water was used throughout this work.

Characterization

Transmission electron microscopy (TEM) observations were performed on a FEI Talos F200S. XPS were carried out with Thermo Scientific KAlpha+. Fourier transform infrared (FT-IR) spectra were obtained on a Nicolet 6700 FT-IR spectrometer. Fluorescence emission and excitation spectra were measured on a Hitachi F-4700 spectrophotometer at ambient conditions. UV-Vis absorption spectra were recorded on a Cary 5000 UV-Vis spectra photometer. Fluorescence spectra and lifetime decay cures of samples were measured with HORIBA QuantaMater 8000 fluorescence spectrophotometer. Photographs/videos of fluorescence emission were taken using a HUAWEI cellphone (Mate 30) under excitation by a hand-hold UV lamp (365 nm).

Synthesis of CDs

First, transfer fresh bamboo leaves to an electric heating constant-temperature forced-air drying oven. Dry them at 80°C for 0.5 hours to remove moisture. Then grind the dried bamboo leaves into powder using a mortar and pestle to obtain a specified amount of bamboo leaf powder, which will serve as the carbon source for subsequent experiments.

Weigh 0.05 g of bamboo leaf powder into a 100 mL PTFE reactor. Dissolve it in 40 mL of anhydrous ethanol. Place the mixture in an ultrasonic cleaner and agitate for 10 minutes to ensure complete dissolution. Then react the mixture at different temperatures 140°C, 150°C, and 160°C for 8 hours each. Allowing the solutions to cool

naturally to room temperature yielded red, pink, and blue solutions, designated as R-CDs, P-CDs, and B-CDs, respectively.

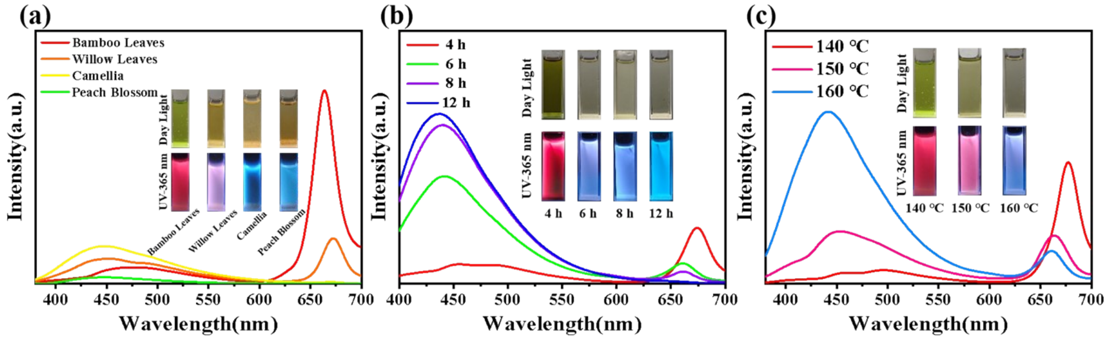

Fig. S1. (a) 365 nm excitation spectra of CDs products synthesized from bamboo leaves, weeping willow, camellia, and peach blossom precursors. Supplementary images show actual specimens under fluorescent light (top) and 365 nm UV light (bottom). (b) 365 nm excitation spectra of CDs products reacted for 8 h at 140 ℃, 150 ℃, and 160 ℃. (c) 365 nm excitation spectra of CDs products obtained after 4 h, 6 h, 8 h, and 12 h reactions at 160 ℃. The figure shows the actual appearance under fluorescent light (top) and 365 nm UV light (bottom).

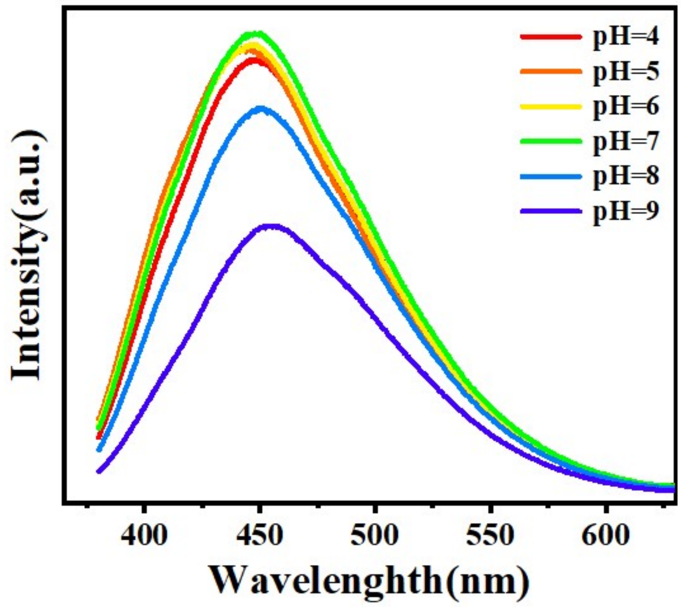

Fig. S2. Fluorescence emission spectra of B-CDs at different pH values.
